# The Effect of Strain Aging on the Microstructure and Mechanical Properties of Steel for Reel-Lay Coiled Steel Pipelines

**DOI:** 10.3390/ma18153462

**Published:** 2025-07-24

**Authors:** Yuxi Cao, Guofeng Zuo, Yang Peng, Lin Zhu, Shuai Tong, Shubiao Yin, Xinjun Sun

**Affiliations:** 1 Faculty of Metallurgical and Energy Engineering, Kunming University of Science and Technology, Kunming 650093, China; 2 Central Iron and Steel Research Institute Group, Engineering Steel Institute, Beiing 100081, China; 3 Hengyang Valin Steel Tube Co., Ltd., Hengyang 421001, China; hgjfzgf@163.com (G.Z.); zhulinling@126.com (L.Z.)

**Keywords:** reel-lay pipeline steel, strain aging, work hardening rate, tempering temperature, dislocation configuration evolution

## Abstract

Deep-sea oil and gas pipelines undergo significant plastic strain during reel-lay installation. Additionally, the static strain aging phenomenon that occurs during service can further deteriorate the mechanical properties of the pipelines. This study investigates the plastic deformation mechanism of reel-lay pipeline steel by subjecting the test steel to 5% pre-strain followed by aging treatment at 250 °C for 1 h. The present study systematically correlates the evolution of mechanical properties with microstructural changes through microstructural characterization techniques such as EBSD, TEM, and XRD. The results demonstrate that after pre-straining, the yield strength of the experimental steel increases due to dislocation strengthening and residual stress generation, while its uniform elongation decreases. Although no significant changes in grain size are observed macroscopically, microstructural characterization reveals a substantial increase in dislocation density within the matrix, forming dislocation cells and walls. These substructures lead to a deterioration of the material’s work hardening capacity. Following aging treatment, the tested steel exhibits further increased yield strength and reduced uniform elongation. After aging treatment, although the dislocation density in the matrix slightly decreases and dislocation tangles are somewhat reduced, the Cottrell atmosphere pinning effect leads to a further decline in work hardening capability, ultimately resulting in the deterioration of plasticity in reel-lay pipeline steel. The instantaneous hardening exponent curve shows that the work hardening phenomenon becomes more pronounced in the tested steel after strain aging as the tempering temperature increases.

## 1. Introduction

Subsea pipelines are an indispensable component in offshore oil and gas exploration and transportation, and are widely recognized as the most efficient and cost-effective method for subsea hydrocarbon transportation [1,2]. Currently, there are three mainstream pipeline installation methods worldwide: the J-lay method, S-lay method, and reel-lay method [3,4]. The most significant difference between the reel-lay method and the other two pipelaying methods is that it allows prefabrication operations such as welding, quality inspection, and coating protection to be performed onshore, which greatly improves work efficiency. The reel-lay method achieves an average laying speed of up to 1 km/h and reduces costs by approximately 30% compared to the other two installation methods, making it the most economical and efficient solution for deepwater and ultra-deepwater pipeline installation [5,6]. However, since the reel-lay method requires pipelines to undergo coiling, uncoiling, and straightening processes, it generates significantly more nonlinear plastic deformation compared to the other two installation methods [7,8]. Pipelines subjected to plastic strain will also experience static strain aging effects during service. Therefore, the reel-lay method imposes higher requirements on the plasticity of pipelines.

Numerous studies have been conducted on reel-lay pipeline steels to date. Plastic strain induces the gradual glide of dislocations along slip planes within the pipeline matrix. During dislocation slip, multiplication and rearrangement also occur, forming spatially periodic dislocation configurations such as dislocation tangles, dislocation walls, and dislocation cells [9,10]. The interaction between adjacent dislocations intensifies due to dislocation multiplication, resulting in dislocation strengthening within the pipeline. Dislocation strengthening leads to increased yield strength but reduced work hardening capacity in pipelines, consequently diminishing their overall plastic deformation capability [11,12,13,14]. The residual strain generated inside the pipeline due to plastic deformation can also severely degrade the fatigue performance of the pipeline and reduce its service life [15,16]. During long-term subsea service, the pipeline undergoes static strain aging due to the pinning effect of Cottrell atmospheres, leading to further deterioration of its mechanical properties [17,18]. Strain aging will further increase the yield strength of the pipeline, causing the yield-to-tensile ratio to approach 1 and reducing its plastic deformation capacity [19,20]. Simultaneously, it elevates the ductile-to-brittle transition temperature (DBTT) of the pipeline, thereby increasing the risk of fracture during service [21].

Extensive research has been conducted on steels for reeled pipelines to date. Research by Yan-Hua Li et al. [22]. on the microstructure and chemical composition of X90 pipeline steel revealed that the type and proportion of microstructural constituents have a far greater impact on service performance than chemical composition. Notably, X90 steel with a dual-phase microstructure was found to possess a significantly higher strain aging susceptibility coefficient. Hyo Kyung Sung et al. [23] found that when the volume fraction of acicular ferrite (AF) increases and the second phase is uniformly distributed in API X60 pipeline steel, the steel exhibits minimal susceptibility to strain aging. Research by Hidenori Shitamoto et al. [24] revealed that the changes in tensile properties of pipelines after strain aging are direction-dependent relative to the pre-strain orientation: yield strength increases when the directions are aligned, but decreases when they are opposed. However, regardless of directional alignment, the plastic deformation capacity of the pipeline undergoes significant deterioration. Nagai et al. [25] achieved dual-phase steels composed of ferrite and bainite with varying ratios by controlling the contents of C, Mn, and Nb followed by heat treatment processes, enabling reel-lay pipeline steels to obtain lower yield ratio and excellent work hardening capability.

In summary, current research on reel-lay pipeline steels primarily focuses on tensile-compressive cyclic plastic strain, with the strain magnitude predominantly concentrated within the range of 1–3% [26,27]. However, during the reel-lay installation process, the maximum single-cycle strain imposed on pipelines can reach up to 5%. Currently, research on steel for Reel-lay pipelines primarily employs finite element simulation to analyze the stress conditions of the pipe during the laying process [8,28,29]. This method cannot systematically analyze the microstructural changes in the coiled pipe.In this context, the present study systematically characterizes microstructural evolution during strain aging using electron backscatter diffraction (EBSD) and transmission electron microscopy (TEM), elucidating in detail the mechanism by which strain aging affects the plasticity of reel-lay pipeline steel.

## 2. Experimental Materials and Methods

### 2.1. Experimental Materials

**Figure 1:**
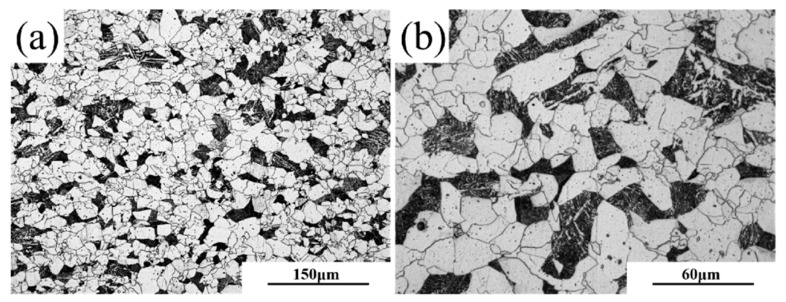
Original microstructure metallographic diagram of experimental steel at (**a**) 200× and (**b**) 500×.

The experimental material employed in this study was laboratory-fabricated X65-grade steel plate with a thickness of 30 mm. The chemical composition of the experimental steel is shown in Table 1. Thermodynamic2023b software calculations indicated that the experimental steel began austenitization at 711.3 °C and completed austenitization at 847.5 °C. The metallographic image of the original microstructure of the experimental steel is presented in Figure 1. To address the issues of non-uniform microstructure and excessive grain size in the experimental steel, this experiment first conducted a full quench at 900 °C to refine the grains. Subsequently, a subcritical quench was performed at 830 °C to form an appropriate ratio of ferrite and martensite within the matrix. Finally, to investigate the effect of high-temperature tempering on the uniform elongation of the experimental steel before and after strain aging, high-temperature tempering was carried out at 650 °C and 710 °C, respectively.

### 2.2. Strain Aging Experiment

**Figure 2:**
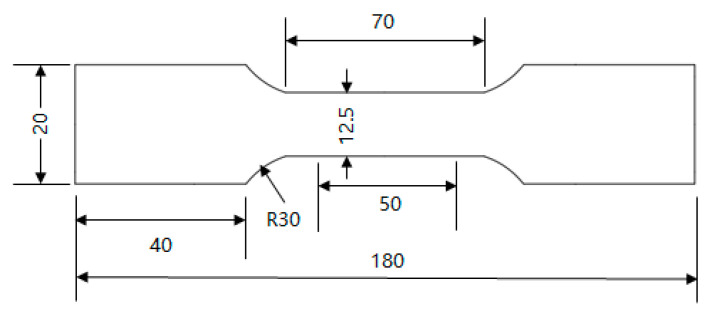
Dimensional diagram of plate-like tensile specimen.

The tensile specimens in this experiment were plate-shaped tensile specimens specified by Standard GB/T 228-2021 [30], with specific dimensions shown in Figure 2. The plate tensile specimens were pre-stretched using an Instron-5958 (Instron, Norwood, MA, USA) universal electronic testing machine. The entire process employed strain control, with an axial electronic extensometer utilized to monitor and regulate the specimen’s deformation. The tests were conducted at 23 °C with a strain rate of 0.00025/s. The specimens were unloaded from the testing machine upon reaching 5% deformation.

According to the DNV-OS-F101 standard [31], the specimens pre-strained by 5% underwent artificial aging treatment at 250 °C for 1 h. The aging treatment was conducted using a box-type tempering furnace at 250 °C with a holding time of 1 h, followed by air cooling.

### 2.3. Mechanical Performance Experiment

The tensile tests were conducted in accordance with the national standard GB/T 228-2021 “Metallic materials—Tensile testing—Part 1: Method of test at room temperature” [30]. The instrument used is the Instron-5958 (Instron, USA) universal electronic testing machine. The test temperature was maintained at 25 °C. A strain-controlled tensile rate of 0.00025/s was applied until yield completion, followed by displacement control at 0.0067/s. The entire process was monitored using an extensometer.

The hardness test was performed on the experimental steel using a VH-5V Vickers hardness tester in accordance with Standard GB/T 4340.1-2024 “Metallic materials—Vickers hardness test—Part 1: Test method” [32], with an applied load of 10 kg and a dwell time of 10 s.

### 2.4. Microstructure Characterization

The experimental steel was sectioned into 10 × 10 × 10 mm cubic specimens, followed by surface grinding and polishing using abrasive papers and polishing machines. The specimens were then etched with 4% nitric acid alcohol solution. Microstructural characterization was performed using an Olympus GX51 (Olympus Corporation, Tokyo, Japan) optical microscope and an FEI Quanta 650FEG (Thermo Fisher Scientific, Waltham, MA, USA) thermal field-emission scanning electron microscope (SEM).

For EBSD characterization, the polished specimens were subjected to electropolishing. A 4% perchloric acid solution served as the electrolyte, with liquid nitrogen added to cool the solution to −10 °C. Electropolishing was conducted at 25 V for 20 s. EBSD analysis was performed using an Oxford F-plus (Oxford Instruments, Abingdon, UK) electron backscatter diffraction detector integrated with a field-emission scanning electron microscope (FE-SEM). The acquired EBSD data were processed using HKL-Channel 5 2019 v5.12 (Oxford Instruments NanoAnalysis, Oxford Instruments, Abingdon, UK) and AztecCrystal 2.1.2 (Oxford Instruments NanoAnalysis, Oxford Instruments, Abingdon, UK) software to low/high-angle grain boundary maps, and kernel average misorientation (KAM) maps.

For XRD analysis, the mechanically polished specimens were subjected to electrochemical etching. The etching solution was prepared by mixing distilled water, potassium chloride, and citric acid in a 50:5:1 ratio. The process was conducted at 0.8 V with a controlled current of 0.08 mA for approximately 20 s. Phase characterization was performed using a D8 ADVANCE X-ray diffractometer (Bruker Corporation, Billerica, MA, USA), and the dislocation density was calculated from the obtained diffraction patterns.

The microstructure of the experimental steel was observed using the thin-film method in this study. The section thickness was kept below 5 μm. Ion thinning was performed using an electrolytic twin-jet polisher, with the electrolytic twin-jet solution being a 6% perchloric acid solution, at a test temperature of −20 °C. The thinned slices were then observed using an FEI TECNAI G2 F20 (FEI Company, Hillsboro, OR, USA) transmission electron microscope (TEM) to examine the microstructural morphology of the test steel.

## 3. Experimental Results

### 3.1. Mechanical Properties Before and After Strain Aging

The steel sample tempered at 650 °C was designated as Steel-650, and the one tempered at 710 °C was labeled as Steel-710. Table 2 shows the mechanical properties of Steel-650 and Steel-710 before and after strain aging. After pre-stretching, both Steel-650 and Steel-710 exhibited significant increases in yield strength (Rp_0.2_)—by 99.5 MPa and 110 MPa, respectively. However, the tensile strength (Rm) showed relatively smaller increments of 21.5 MPa and 37.5 MPa after pre-straining. Following aging treatment, the yield strength further increased (65 MPa for Steel-650 and 44 MPa for Steel-710), while the tensile strength remained essentially unchanged, resulting in yield-to-tensile ratios approaching 1. The uniform elongation (Agt) values decreased by 4.5% and 4.75% for Steel-650 and Steel-710, respectively, after pre-straining. Post-aging, Steel-650’s Agt approached zero, while Steel-710’s uniform elongation further decreased by 2.01%. 

**Figure 3:**
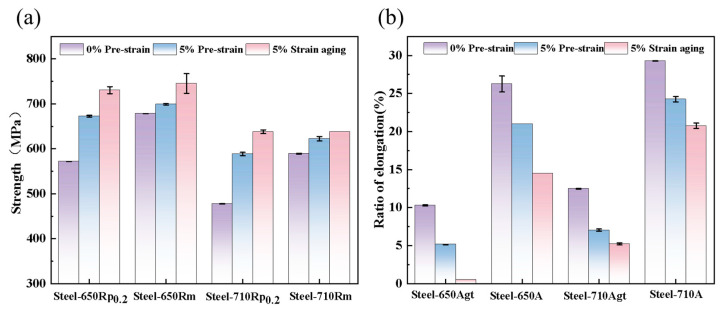
Steel-650 and Steel-710 comparison of mechanical properties of steel before and after strain aging: (**a**) strength comparison; (**b**) elongation comparison.

As can be seen from Figure 3a, the increase in yield strength of Steel-650 after pre-straining and aging treatment was smaller than that of Steel-710. Figure 3b indicates that after 5% pre-deformation and aging treatment, the reduction in Agt was significantly less than that in elongation after fracture (A). This proves that the detrimental effect of strain aging on plastic deformation mainly occurs during the uniform plastic deformation stage, while having relatively little influence on the non-uniform deformation stage from necking to fracture.

### 3.2. Microstructural Morphology Before and After Strain Aging

**Figure 4:**
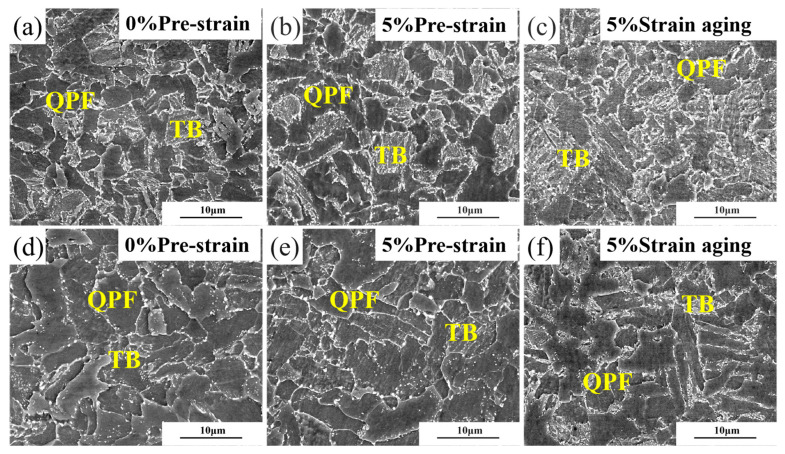
Microstructure morphology before and after 5% strain aging: (**a**–**c**) Steel-650; (**d**–**f**) Steel-710.

To compare the microstructural changes of the experimental steel before and after strain aging, this experiment observed the experimental steel using a scanning electron microscope. Figure 4 presents the microstructures of Steel-650 and Steel-710 before and after 5% strain aging. As shown in Figure 4a,d, the tempered steel microstructure consisted of quasi-polygonal ferrite (QPF) and tempered bainite (TB). In Steel-650, the TB retained the original martensitic lath structure, with granular cementite particles dispersing along grain boundaries and subgrain boundaries. When the tempering temperature was increased to 710 °C (Figure 4d), the lath structure within TB disappeared and the grains gradually became equiaxed. Figure 4b,e demonstrates that after 5% pre-deformation, neither grain size nor morphology exhibited significant changes. Following aging treatment (Figure 4c,f), a pronounced increase in cementite precipitation was observed in both steels.

**Figure 5:**
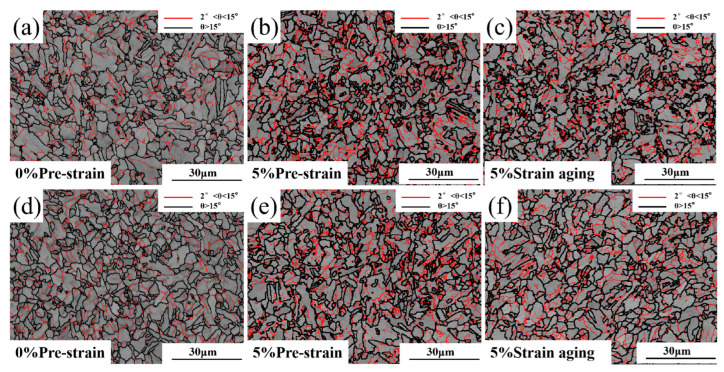
Grain boundary distribution before and after strain aging: (**a**–**c**) Steel-650; (**d**–**f**) Steel-710.

**Figure 6:**
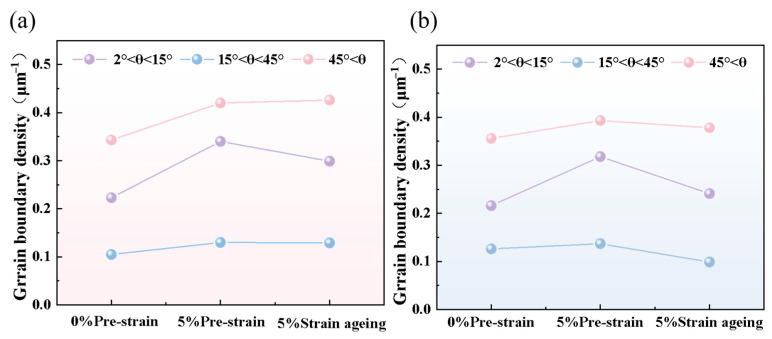
Grain boundary density distribution before and after 5% strain aging: (**a**) Steel-650; (**b**) Steel-710.

The EBSD data were processed using HKL-Channel 5 2019 v5.12 software to obtain the following images. Figure 5 shows the grain boundary distribution before and after 5% strain aging, where grain boundaries with misorientation angles between 2° and 15° are colored red as low-angle grain boundaries (LAGBs), and those with misorientation angles greater than 15° are colored black as high-angle grain boundaries (HAGBs). HAGB mainly reflects the grain boundaries of the matrix structure, while LAGB primarily reflects the substructures and dislocations within the grains. Figure 5d,e shows that the LAGBs significantly increased in the matrix after 5% pre-straining. According to the statistical data in Figure 6, after pre-straining, the HAGB density of Steel-650 increased by 0.115 and the LAGB density increased by 0.118, while for Steel-710, the HAGB density increased by 0.048 and the LAGB density increased by 0.102. Figure 5c,f shows that after aging treatment of the pre-strained steel, the densities of both HAGBs and LAGBs decreased, but remained higher than in the original state. According to the statistical data in Figure 6, after aging, the HAGB density of Steel-650 slightly decreased by 0.001 and the LAGB density decreased by 0.041, while for Steel-710, the HAGB density decreased by 0.064 and the LAGB density decreased by 0.077.

**Figure 7:**
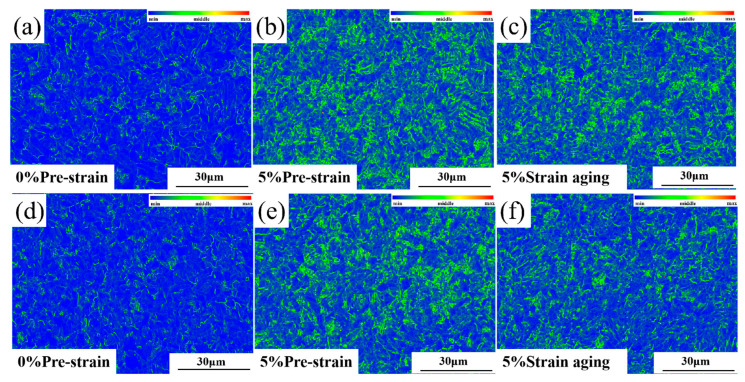
The KAM maps of Steel-650 and Steel-710 before and after 5% strain aging: (**a**–**c**) Steel-650; (**d**–**f**) Steel-710.

The KAM maps reflect the local strain distribution and geometrically necessary dislocation density within the matrix by calculating the average misorientation between adjacent pixels. This allows quantification of local pre-strain levels to evaluate deformation uniformity and reveals stress distribution in the material. The rainbow-colored band in the upper left corner of the KAM image serves as a scale bar. The more green areas present in the image, the more severe the stress concentration within the matrix of the tested steel. Comparing Figure 7a,b,d,e, it can be observed that in non-pre-strained samples, stresses primarily concentrate at grain boundaries and internal substructures. After pre-straining, the overall KAM values in the matrix significantly increase, with stress–strain distribution becoming relatively uniform throughout the material. Stress concentrations no longer appear exclusively at grain boundaries, but predominantly within grain interiors. From the comparison of KAM maps before and after aging (Figure 7b vs. Figure 7c and Figure 7e vs. Figure 7f), the intensity of stress–strain concentrations clearly decrease following aging treatment, though remaining higher than in the original state.

**Figure 8:**
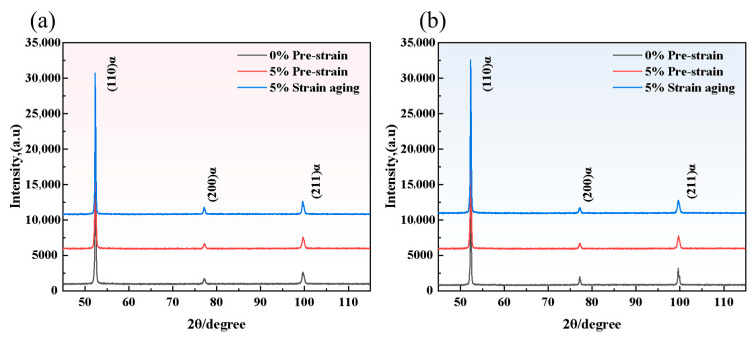
XRD diffraction patterns of Steel-650 and Steel-710 steels before and after strain aging: (**a**) Steel-650; (**b**) Steel-710.

Figure 8 had shown the X-ray diffraction patterns of Steel-650 and Steel-710 after strain aging. The dislocation density had been calculated using information from the diffraction peaks in the patterns, with the calculation results shown in Table 3. The dislocation density in the matrix of Steel-650 in the original state was 1.01108 × 10^10^, which had increased to twice the previous value at 2.1824 × 10^10^ cm^−2^ after 5% pre-straining, and had then decreased slightly to 1.8174 × 10^10^ cm^−2^ after aging treatment. Due to the increased tempering temperature, the dislocation density in the matrix of Steel-710 in the original state was lower at 8.5295 × 10^8^ cm^−2^, which had increased exponentially to 1.5864 × 10^10^ cm^−2^ after pre-straining, and had then decreased to 1.0854 × 10^10^ cm^−2^ after aging treatment.

## 4. Discussion

### 4.1. Stress–Strain Response Before and After Strain Aging

**Figure 9:**
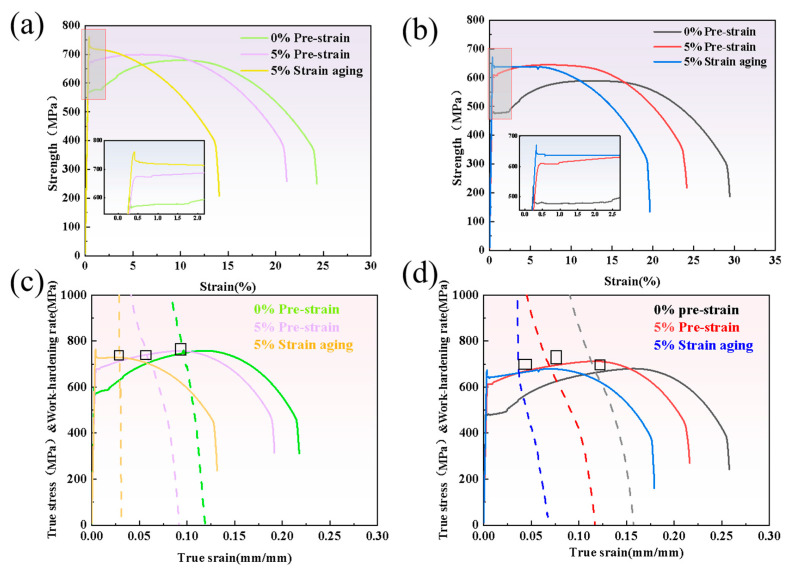
Mechanical property curves of Steel-650 and Steel-710 before and after strain aging: (**a**,**b**) engineering stress–strain curves of Steel-650 and Steel-710 before and after strain aging; (**c**,**d**) true stress–strain curves and work hardening rate curves of Steel-650 and Steel-710 before and after strain aging (the dashed line represents the work hardening rate curve).

The engineering stress–strain curves in Figure 9a,b show that the specimens without pre-strain have obvious yield platforms, as well as upper yield points and lower yield points during the tensile process. During the yielding stage of carbon steel under tensile loading, dislocations within the matrix gradually overcome the pinning effects of interstitial atoms (such as carbon and nitrogen) under applied stress. These solute atoms are commonly referred to as Cottrell atmospheres, which predominantly distribute around dislocations to release lattice distortion energy or atomic-scale microstresses [33,34]. Due to the strong pinning effect of Cottrell atmospheres on dislocations, dislocations can only break away from Cottrell atmosphere pinning under relatively high external stress, and this stress value corresponds to the upper yield point in the engineering stress–strain curve.

After 5% pre-strain, the engineering stress–strain curve of the specimen shows disappearance of the upper yield point, significant shortening of the serrated yield plateau, and a pronounced increase in yield strength. This is because during the 5% pre-straining process, the dislocations in the matrix have already overcome the pinning effect of Cottrell atmospheres and become mobile dislocations, resulting in the disappearance of the upper yield point and overall shortening of the yield stage in the stress–strain curve. The reasons for the increase in yield strength after pre-strain mainly fall into two categories. The first reason is that the pre-straining process induces significant dislocation multiplication, resulting in dislocation strengthening of the test steel, which requires higher external stress to initiate plastic deformation [35]. The second reason is that after plastic deformation occurs in a specific direction, dislocation multiplication and rearrangement take place within the metal matrix. This leads to inhomogeneous distribution of dislocations across grain interiors. During further straining, incompatibility arises between regions of high dislocation density and those of low dislocation density. Such incompatibility generates residual stresses opposing the deformation direction at the grain scale, thereby enhancing the material’s resistance to further plastic flow [36]. Furthermore, during the pre-straining process, to ensure compatibility of plastic deformation across grain boundaries, adjacent grains rotate in opposite directions during plastic straining. This coordinated rotation enables synchronous deformation of all grains, ultimately resulting in increased misorientation angles between grains, higher density of HAGB, and elevated intergranular stresses. Therefore, considering all the above factors, both intragranular and intergranular stresses generated during pre-deformation will collectively lead to an increase in the material’s yield strength.

Upon aging at 250 °C for 1 h, the pre-deformed specimens demonstrated a further enhancement in yield strength, with the upper yield point reappearing in their stress–strain curves. This occurs because during the aging process, carbon and nitrogen atoms originally dissolved in the matrix become more active due to thermal activation, migrating toward adjacent dislocations and reforming stable Cottrell atmospheres [37].

The pre-straining process generates a high density of dislocations in the experimental steel, which provides numerous nucleation sites for Cottrell atmosphere formation. Consequently, higher external stress is required to enable dislocations to overcome this pinning effect and initiate slip. Previous studies have demonstrated that the intersection point between the true stress–true strain curve and the work hardening rate curve marks the onset of necking in materials. Prior to the necking stage, the material undergoes uniform plastic deformation [35]. As seen in Figure 9c,d, after 5% pre-strain and aging treatment, the intersection point between the work hardening rate curve and true stress–true strain curve shifts significantly forward. Compared to non-pre-strained steel, the 5% strain-aged steel exhibits weaker plastic deformation capability, shorter uniform plastic strain stage, and earlier necking initiation point, resulting in reduced pipeline plasticity.

### 4.2. Evolution of Dislocation Morphology

**Figure 10:**
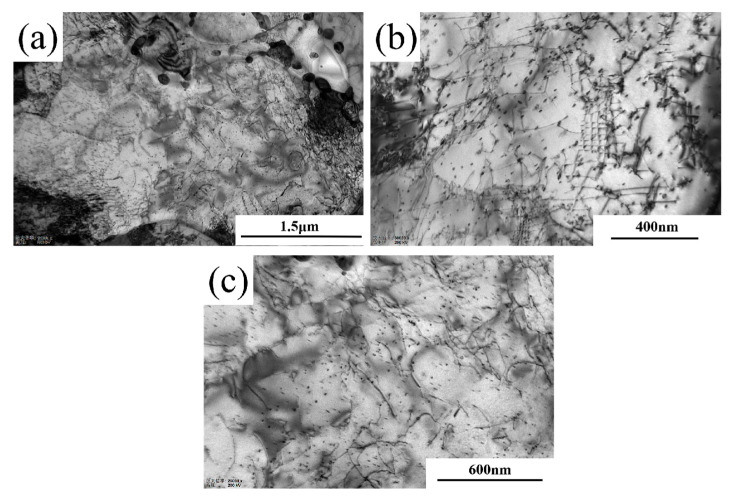
Dislocation morphology of Steel-710 before strain aging. (**a**) 12,000×; (**b**) 30,000×; (**c**) 25,000×.

**Figure 11:**
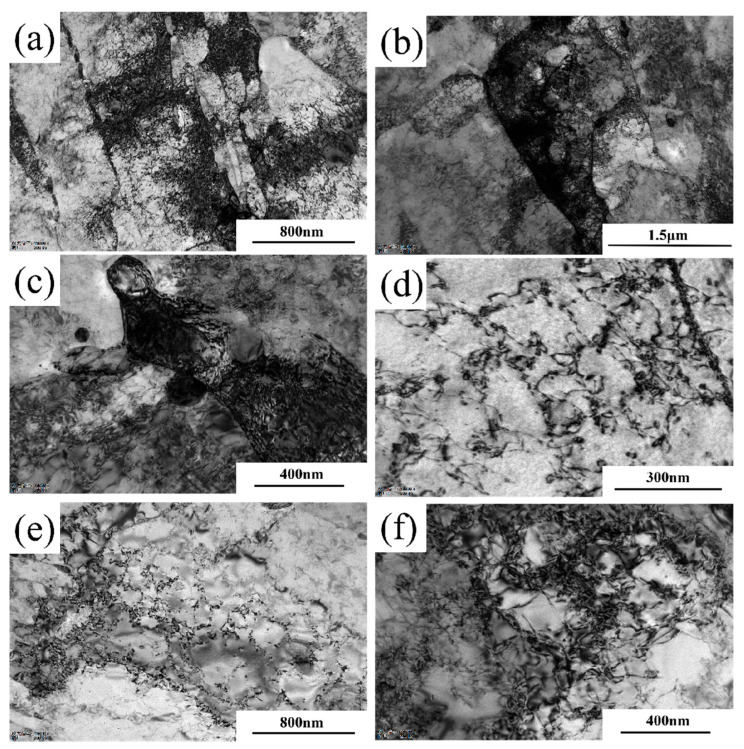
Dislocation morphology of Steel-710 after 5% pre-straining. (**a**) 15,000× (**b**) 12,000×; (**c**) 30,000×; (**d**) 50,000×; (**e**) 15,000×; (**f**) 30,000×.

XRD analysis cannot accurately characterize the influence of dislocation configuration evolution on material plasticity. Therefore, this study employs TEM characterization to investigate the strain aging-induced changes in dislocation morphology, using Steel-710 as a representative example. As shown in Figure 10, prior to the 5% strain aging treatment, the dislocations within Steel-710 exhibited relatively sparse entanglement and uniform distribution, primarily existing as discrete dislocation lines. Figure 11 displays the dislocation morphology after 5% pre-straining. As shown in Figure 11a, a large number of high-density dislocation tangles with extremely non-uniform distribution were observed after pre-deformation. The high-density dislocation tangles were mainly found at grain boundaries, as seen in Figure 11b. Figure 11c shows that the alloy cementite at grain boundaries, as hard phases in the matrix with higher strength and hardness, can strengthen the grain boundaries, making them more stable under external forces. According to Figure 11d, since the second-phase particles in the matrix hinder dislocation slip, they cause dislocations to form dislocation loops around these particles [38]. When the deformation increases further, dislocations not only move along a single slip system, but also form multiple slip systems. The formation of multiple slip systems occurs because when the deformation reaches a certain level, the slip planes rotate, and dislocation slip occurs simultaneously on multiple slip systems. When dislocations on different slip systems interact, they form dislocation walls, as shown in Figure 11e. With continued increase in deformation, the dislocation density further increases, and dislocation walls on adjacent slip systems become entangled to form dislocation cells (Figure 11f). During subsequent deformation, dislocations tend to accumulate at dislocation walls and cell walls, leading to increasingly non-uniform dislocation distribution and reduced mobile dislocations. This results in a severe decrease in the uniform deformation capacity of the tested steel and a deterioration of material plasticity.

**Figure 12:**
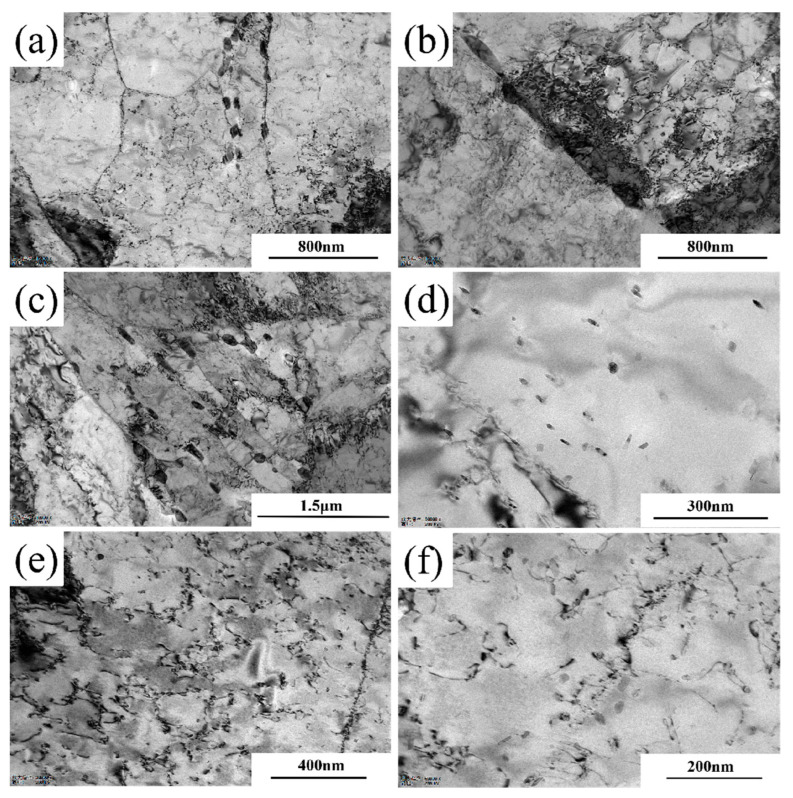
Dislocation morphology of Steel-710 after aging treatment. (**a**) 15,000× (**b**) 15,000×; (**c**) 12,000×; (**d**) 50,000×; (**e**) 30,000×; (**f**) 60,000×.

Figure 12 shows the dislocation configuration of Steel-710 after aging treatment. As seen in Figure 12a, the dislocation density inside the aged sample significantly decreased. The high-density dislocation tangles at grain boundaries were partially relieved, though some remnants remained (Figure 12b). Meanwhile, the number of alloy cementite particles at grain boundaries noticeably increased with larger particle sizes, and precipitation also occurred at subgrain boundaries (Figure 12c). Since there is a significant hardness difference between alloy cementite and the matrix, excessive alloy cementite at grain boundaries can easily lead to stress concentration, increasing the likelihood of crack initiation. Figure 12e,f reveals that during the aging process, the wall thickness of dislocation cells and dislocation walls formed during pre-straining substantially reduced. Dislocation loops around precipitates gradually transformed into dislocation lines, leading to more homogeneous dislocation distribution overall. This primarily occurs because elevated aging temperatures increase the thermal activation energy of dislocations, enabling them to break away from highly tangled configurations and become mobile dislocations. These mobile dislocations interact with other dislocations during slip through processes like dislocation annihilation and cross-slip, thereby reducing the overall dislocation density in the matrix. However, due to the relatively low aging temperature and the strong pinning effect of Cottrell atmospheres formed during aging, the dislocation density does not fully recover to its pre-strain-aged state.

### 4.3. Effect of Strain Aging on Work Hardening Behavior

**Figure 13:**
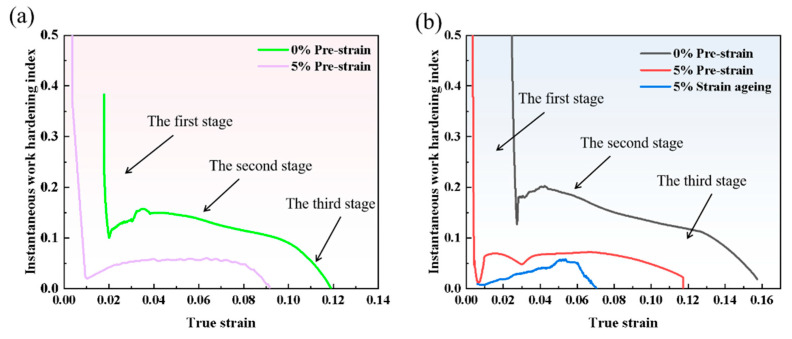
Relationship curves between instantaneous work hardening exponent n and true strain before and after strain aging: (**a**) 1# steel; (**b**) Steel-710 steel.

The work hardening rate represents the rate at which the strength of a material increases with the degree of strain during plastic deformation. An increase in work hardening rate indicates that the metallic material can more rapidly enhance its resistance to deformation during processing. This effectively prolongs the uniform plastic deformation stage and delays the onset of localized strain concentration. The instantaneous work hardening exponent n reflects the rate of change in a material’s ability to resist further plastic deformation during the deformation stage and serves as an important indicator for analyzing the strain hardening behavior of materials. N can more accurately describe the work hardening behavior of a material during the deformation process. The Hollomon empirical equation can be applied to describe materials undergoing uniform deformation. According to (1) Hollomon’s formula, it can be known that [39,40]:

(1) $$\sigma_{true}=K\times \varepsilon_{true}^{n(s,\sigma )}$$

where σ_true is the true stress in MPa, ε_true is the true strain, and K is the material’s work hardening coefficient. Taking the logarithm and derivative of both sides of the equation yields the following expression:

(2) $$n(\varepsilon ,\sigma )=\frac{d\left(ln\sigma_{ture}\right)}{d\left(ln\varepsilon_{true}\right)}=\frac{\varepsilon_{true}}{\sigma_{true}}\times \frac{d\sigma_{true}}{d\varepsilon_{true}}=\frac{\varepsilon_{true}}{\sigma_{true}}\times \theta$$

where θ is the work hardening rate. The curve of instantaneous work hardening exponent versus true strain, obtained by substituting the true stress, true strain, and work hardening rate into the formula, is shown in Figure 13.

As shown in Figure 13, the work hardening behavior of the tested steel can be divided into three main stages. The first stage is the rapid hardening stage, during which the steel transitions from elastic deformation to plastic deformation. The curve reveals that this stage exhibits a high n-value, but the n-value shows a rapid decreasing trend with increasing true strain. Comparing the curves before and after pre-strain reveals that the work hardening behavior occurs later in the non-pre-strained specimen. This occurs because non-pre-strained specimens exhibit yielding behavior during the initial deformation stage, resulting in delayed work hardening. In contrast, the 5% pre-strained specimens already contain numerous dislocations internally. Under applied stress, these dislocations immediately slip, causing interactions and pile-ups with neighboring dislocations, thereby producing work hardening effects more rapidly. Due to the formation of microstructures such as dislocation cells and walls during pre-straining, mobile dislocations become entangled at cell walls and transform into immobile dislocations, leading to a shorter rapid hardening stage in pre-strained specimens.

The second stage is the steady hardening stage, where the change in n-value gradually stabilizes. Comparing the curves before and after pre-straining shows that the n-value after pre-straining is lower than in the original state. This is because the dislocation density within the matrix is already relatively high after pre-straining, resulting in less dislocation multiplication during subsequent deformation and a lower instantaneous work hardening exponent. For specimens without pre-straining, the initial dislocation density in the matrix is lower. After the yield stage, dislocations begin to move and multiply, leading to higher work hardening capacity in this stage compared to the 5% pre-strained specimens. A comparison of the n-values between the non-pre-strained specimens (Steel-650 and Steel-710 in their original states) reveals that the n-value of 1# test steel is lower. This is mainly because 1# steel has a relatively higher dislocation density and contains lath structures with high-density dislocations, both of which limit work hardening behavior during deformation.

The third stage is the work hardening exponent decline stage, where the n-value decreases rapidly with increasing strain. This occurs due to massive dislocation pile-ups at grain boundaries causing stress concentration. When the internal and external stresses exceed the grain boundaries’ capacity, necking begins in the specimen and work hardening behavior disappears.

Comparing Figure 13a,b reveals that the 1# steel no longer exhibits work hardening after aging treatment, while the Steel-710 steel still shows slight work hardening. This is because the tempering temperature of 1# steel is lower than that of Steel-710 steel, and its internal lath structure restricts dislocation movement. After strain aging, the dislocations become highly entangled and, combined with the pinning effect of Cottrell atmospheres, mobile dislocations disappear, resulting in the absence of work hardening.

In conclusion, strain aging significantly affects the material’s work hardening behavior during deformation, severely impairing its uniform plastic deformation capability and reducing its plastic deformation capacity.

## 5. Conclusions

After pre-straining, the yield strength of the experimental steel increases, the yield stage in the stress–strain curve shortens, and the upper yield point disappears. After subsequent aging treatment, the yield point reappears and the yield strength increases again. Following strain aging, the intersection point between the work hardening rate curve and the true stress–strain curve gradually shifts forward, indicating that necking occurs progressively earlier.

EBSD characterization reveals that after pre-straining, the test steel exhibits significantly increased densities of both HAGBs and LAGBs, along with elevated KAM values. Following subsequent aging treatment, these parameters show a slight decrease but fail to return to their original state.

After pre-straining, the dislocation density within the matrix increases, forming highly dense dislocation tangles and creating substructures such as dislocation cells and walls within the grains. Following aging treatment, the dislocation density decreases to some extent, and the dislocation arrangement gradually becomes more uniform.

After strain aging, the work hardening phenomenon disappears in test Steel-650, while test Steel-710 still demonstrates noticeable work hardening behavior. The work hardening effect becomes more pronounced with increasing tempering temperature following strain aging.

## Figures and Tables

**Table 1:** Chemical composition of laboratory-produced X65 steel plate (mass%).

	Fe	C	Si	Mn	Ni	Cr	Mo	V	Nb	Ti	Al
X65	Bal.	0.138	0.25	0.65	0.51	0.46	0.38	0.083	0.021	0.018	0.056

**Table 2:** Steel-650 and Steel-710 mechanical properties of experimental steel before and after strain aging.

	Condition of the Test Steel	Rp_0.2_/Mpa	Rm/Mpa	Rp_0.2_/Rm	Agt/%	A%	HV (10 kg)
Steel-650	0% Pre-strain	571.5	678.5	0.84	10.235	25.5	221.23
		572	678	0.84	10.34	27	
	5% Pre-strain	671	700	0.97	5.12	21	254.3
		674	698	0.97	5.15	21	
	5% Strain aging	730	730	1	0.5	14.5	231.2
		725	761	0.95	0.5	14.5	
Steel-710	0% Pre-strain	477	588.5	0.81	12.505	29.25	193.1
		478	590	0.81	12.43	29.3	
	5% Pre-strain	591	626	0.94	7.13	24.5	224.3
		586	619	0.94	6.91	24	
	5% Strain aging	635	638	0.99	5.12	21	210.6
		641	641	1.00	5.32	20.5	

**Table 3:** Dislocation densities (cm^−2^) of Steel-650 and Steel-710 before and after strain aging.

Steel Grade	0% Pre-Stretching	5% Pre-Stretching	5% Strain Aging
Steel-650	1.01108 × 10^10^	2.1824 × 10^10^	1.8174 × 10^10^
Steel-710	8.5295 × 10^8^	1.5864 × 10^10^	1.0854 × 10^10^

## Data Availability

The original contributions presented in this study are included in the article. Further inquiries can be directed to the corresponding authors.
